# Results from a malaria indicator survey highlight the importance of routine data capture in high-risk forest and farm transmission sites in Vietnam to tailor location-specific malaria elimination interventions

**DOI:** 10.1371/journal.pone.0250045

**Published:** 2021-04-16

**Authors:** Thang Duc Ngo, Sara E. Canavati, Dang Viet Dung, Thuan Huu Vo, Duong Thanh Tran, Long Khanh Tran, Rosalie J. Whedbee, Ekaterina I. Milgotina, Gerard C. Kelly, Kimberly A. Edgel, Nicholas J. Martin

**Affiliations:** 1 Department of Epidemiology, National Institute of Malariology, Parasitology and Entomology, Hanoi, Vietnam; 2 Vysnova Partners, Inc., Landover, Maryland, United States of America; 3 Burnet Institute, Melbourne, Victoria, Australia; 4 Global Scientific Solutions for Health, Baltimore, Maryland, United States of America; 5 United States Naval Medical Research Unit TWO, Singapore; Instituto Rene Rachou, BRAZIL

## Abstract

In-line with the World Health Organization’s (WHO) Global Technical Strategy for Malaria (2016–2030), Vietnam is striving to eliminate malaria by 2030. Targeting appropriate interventions in high-risk populations such as forest and forest-fringe communities is a critical component of malaria elimination efforts in Vietnam. In 2016, a household-level malaria indicator survey was conducted in Phu Yen Province, Vietnam with the aim of assessing the knowledge, behaviors and associated risks of malaria infection among priority mobile and migrant populations (MMPs) working and sleeping in forests and on farms. A total of 4211 people were included in the survey, comprised of 1074 heads of households and 3137 associated household members. Of the 1074 head-of-household respondents, 472 slept in a forest, 92 slept on a farm, 132 slept in both forests and farms, and 378 slept at their villages within the last 12 months. Age, literacy, and occupation were significantly different among those who slept in a forest versus on a farm. Of 301 respondents who answered questions about malaria risk factors at sleeping sites, 35% were somewhat aware of malaria prevention practices, but only 4% could recall at least four malaria prevention messages. Among the same group of 301 respondents, only 29% used nets and only 11% used treated nets. Ownership and use of nets among forest-goers was significantly lower than those who slept on a farm or in their village. Huts without walls were significantly prominent forest sleeping site locations (POR = 10.3; 95% CI 4.67–22.7). All respondents who slept in a forest requested standby malaria drugs and one-third of them self-treated without blood testing. Results from this study highlight the importance of capturing relevant location-specific data among priority populations such as remote forest and farm going mobile and migrant populations in Vietnam. Data regarding behavioral practices, knowledge, preventative measures, and intervention coverage at remote-area transmission sites must be routinely captured to effectively monitor progress and refine targeted intervention strategies accordingly.

## Introduction

As a result of the increased emergence of drug resistant malaria in the late 1980’s, Vietnam’s National Malaria Program shifted its focus to intensify and expand malaria control efforts throughout the country [1]. Between 1991 and 2010, Vietnam experienced a 95% drop in malaria cases and a 99.5% reduction in malaria deaths, with no locally-acquired cases reported in 13 of the 58 provinces in Vietnam (22%) [2–5]. Due to this significant reduction in the malaria burden, Vietnam has since reoriented its program towards malaria elimination, with a goal of eliminating local transmission by 2030.

The Vietnam National Institute of Malariology, Parasitology and Entomology’s (NIMPE) National Strategy for Malaria Control and Elimination 2011–2020 and Orientation to 2030 outlines current national objectives and a strategic roadmap to achieve elimination [6]. Key strategies include: (i) ensuring all people have better access to early and effective malaria diagnosis and treatment at public and private health facilities; (ii) ensuring the optimal coverage of populations at risk of malaria through appropriate control measures; (iii) eliminating malaria in provinces with low endemicity and reducing malaria incidence in high and moderate endemic provinces; (iv) improving malaria epidemiological surveillance systems and ensuring sufficient malaria epidemic response capacity; and (v) improving knowledge of malaria prevention and implementing associated behavior change to empower the people to actively protect themselves from malaria [6].

In line with key global strategies, such as the World Health Organization’s (WHO) Global technical strategy for malaria 2016–2030 [7], a key component of Vietnam’s elimination strategy is to target and ensure optimal intervention coverage in known high transmission risk areas including provinces of the Central and Central-Highlands regions, the Southeast region, and regions bordering China (Yunnan Province), Laos and Cambodia [6]. Populations within these key regions are large, with more than 15 million people living Vietnam’s mountainous regions, coastal areas or on remote borders. Vietnam’s forested and mountainous areas also attract many migrant workers from non-malaria endemic provinces and from the border areas of Laos and Cambodia [8].

Vietnam faces a number of significant epidemiological complexities that characterize malaria transmission in these priority regions. Major *Anopheles* species found in the forested areas of South-Central Vietnam include *An*. *dirus*, *An*. *maculatus s*.*l*., and *An*. *minimus s*.*l*. [9–11]. Complexities associated with varying vector-specific feeding traits, resting behavior, and seasonality complicate malaria elimination efforts and present challenges for traditional malaria prevention interventions [12]. For example, the exophilic nature of *An*. *dirus*, *An*. *maculatus s*.*l*. and *An*. *minimus s*.*l*. decrease effectiveness of insecticide indoor residual spraying, while their early evening/early morning exophagic activity diminishes the role of insecticide-treated nets in malaria prevention [9]. Additionally, drug-resistant malaria parasites have also appeared at different rates in many provinces, mostly in the Central Highlands and Southeast areas [13,14]. An increasing prevalence of *Plasmodium vivax* malaria is also changing the dynamics of the malaria elimination program, as *P*. *vivax*’s unique biological cycle (including days-to-years long dormant stage in liver) coupled with the requirement for a longer treatment course may significantly attenuate malaria elimination in Vietnam [15,16].

Between 2009 and 2015, a total of 57,713 *Plasmodium falciparum* and 32,386 *Plasmodium vivax* cases were reported in Vietnam [17]. While the overall burden of malaria and the incidence of *P*. *falciparum* declined during this period, the incidence of *P*. *vivax* increased, rising from 0.3 cases per 10,000, to 0.41 in 2015, with a peak of 0.64 in 2014 [17]. As malaria has declined the spatial distribution of transmission has become more heterogeneous and concentrated to small geographic pockets, such as the forested district of Dong Xuan in Phu Yen Province [18]. These remote forest regions in central Vietnam are synonymous for having high proportions of asymptomatic infections [19], and high numbers of mobile and migrant populations (MMPs) working and sleeping in forest environments [20]. Previous surveys in South East Asia have indicated that high proportions of MMPs do not actively engage in malaria prevention practices, leading to poor intervention coverage and an increased risk of infection [21–23]. These factors highlight the risk of introducing and/or sustaining ongoing malaria transmission through continuous population movement. Without the adoption of appropriate malaria prevention interventions in these environments, threats to malaria elimination exist both in regards to the importation of malaria from other high burden endemic areas, as well exporting malaria to low burden or malaria-free receptive locations. These challenges call for the need to accurately capture essential data in these high priority areas to better understand potential transmission dynamics and to identify gaps and opportunities to support Vietnam’s key elimination strategies.

To provide guidance to the Vietnam National Malaria Program, a household level malaria indicator survey was conducted in Dong Xuan District, Phu Yen Province in 2016. The aims of the study were to compare the risk factors of respondents who slept in a forest versus those who slept on a farm over a 12-month period and to describe malaria prevention knowledge, attitudes, and practices among these groups. Additionally, the study provided the opportunity to assess Vietnam’s malaria elimination progress among high-priority forest or farm sleepers.

## Materials and methods

### Study site

**Fig 1:**
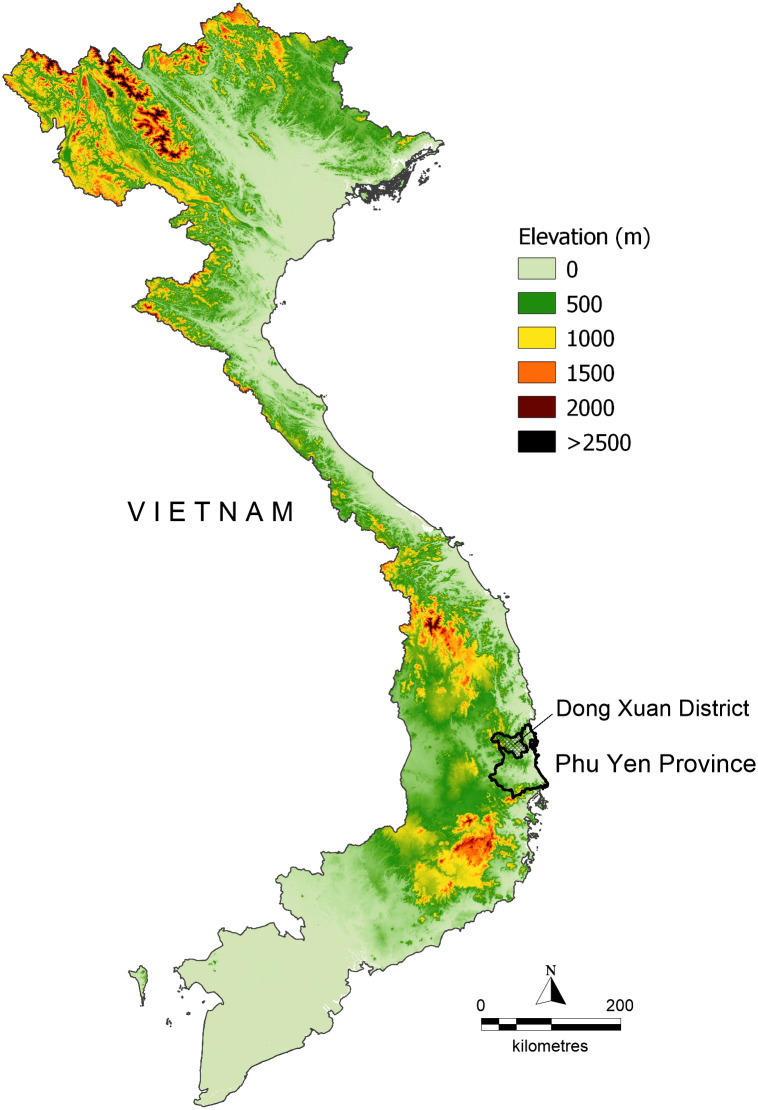
Study site location map highlighting major mountain ranges in Vietnam. Custom map produced by the authors using *MapInfo Professional v15*.*0*.*2* (Pitney Bowes Software Inc. 2015, Stamford, CT; https://www.pitneybowes.com).

The study was conducted in three mountainous communes in Dong Xuan District, located in the south-central coastal province of Phu Yen (Fig 1). The selected communes of Phu Mo, Xuan Lanh, and Xuan Quang 1 represent the three highest malaria-burden communes of Dong Xuan district. This study site was purposefully selected by NIMPE due to its epidemiological characteristics including the relatively high burden of malaria cases, a high number of forest-fringe communities, and large proportions of the local population that frequently travel to and sleep in the forest and/or on farms for their livelihood [24].

### Study design

As part of the development of a targeted geospatial malaria surveillance system in Vietnam, geographical reconnaissance data collection operations were conducted in 2015 to locate and enumerate all households in priority regions, including 4,668 households within the specific study site area of Dong Xuan District, as a means to provide a baseline of households and associated populations residing in these locations [25]. Between August and September 2016, a cross-sectional malaria indicator survey was conducted in the study area, utilizing the available geographical reconnaissance household data as a baseline reference. Of the 4,668 households enumerated in the study site area, 1,083 were randomly selected for inclusion in the survey using their unique household number as an identifier. The survey was conducted using face-to-face interviews with the head of each household to collect information about themselves and their respective associated household members. As part of the survey, household head respondents were asked to self-identify if they slept in forest and/or farm locations within the previous 12 months to group participants into relevant sleeping site location categories, including: slept on a farm; slept in the forest; slept at both sites; or slept at home residence. A template of the survey questionnaire used in the study has been provided in both Vietnamese and English as a Supporting Information File (S1 File).

In instances where the head of the household was not available, the next responsible household member over the age of 18 was interviewed. Individuals that were directly interviewed in the study were labelled as the primary respondents. Within the target area, only those households whose primary respondents were between 18 and 70 years old were included in the survey. Nine primary respondents did not meet the eligibility criteria and were excluded.

### Data collection and analysis

#### Data collection tool

A detailed structured survey tool was developed and included 80 questions relating to demographics, mosquito net use, predominant work practices, forest and farm sleeping site characteristics, risk factors of malaria infection at forest and farm locations, and knowledge and attitudes on malaria prevention measures. The questionnaire was pretested in a site not included in the survey and revised accordingly. Digital data collection forms were developed using a smart-phone application (KLL Collect^®^, Kathmandu Living Labs, Kathmandu, Nepal) and uploaded using a web-based database platform (ONA^®^ Systems, Nairobi, Kenya). Household location coordinates were captured for each interview site and a picture of each net type was taken to confirm accuracy of reporting. Sleeping structures were also inspected at selected external forest and farm locations where malaria transmission was suspected to have occurred, and classified as houses/huts with wall, huts without walls, and hammocks/outdoors.

#### Field data collection

NIMPE staff provided training to local-level district and commune health personnel on study procedures, including selection of households, interview procedures, data collection, and confidentiality of participants. Data were collected by district and commune health officers, and NIMPE personnel using the smartphone application and uploaded in real-time to the web-based platform. Respondents were informed that participation was both voluntary and confidential, with no personal identifiers captured by the interviewers. Efforts were also made to ensure local level health staff (commune and district) with well-established community presence in the study sites led the interview process at all times. Collected data files were reviewed periodically for completeness by field supervisors as part of data quality monitoring activities.

#### Data analysis

Data were analyzed using R software (Epi, car and BMA packages, version 3.1.4 R Foundation). Frequencies and proportions were used to describe categorical variables, and medians were used to describe continuous variables. Chi square was used to assess any statistically significant differences in socio-demographic characteristics between the study groups. Logistic regression models were used to calculate prevalence odds ratios (PORs), adjusted PORs and 95% confidence interval (CI) to assess the association between characteristics among those who slept in forests, on farms and related factors. A *P*-value of <0.05 was considered significant.

### Ethical clearance and consent

The study protocol was reviewed and approved by the National Institute of Malariology, Parasitology and Entomology (NIMPE) Institutional Review board in Hanoi, Vietnam (Protocol number: 2016–VM-P1; IRB Number: IRB00010326; Date: August 10, 2016) and the United States Naval Medical Research Unit TWO (NAMRU-2) Human Research Protection Office (HRPO.NMRCA.2016.0002; Date: August 11, 2016) in compliance with all applicable federal regulations governing the protection of human subjects. Written informed consent was obtained from all study subjects and identities of individuals and establishments were kept confidential.

## Results

Of the 1074 primary household head respondents, 279 (26%) resided in Phu Mo, 392 (36%) in Xuan Quang 1, and 403 (38%) in Xuan Lanh. Among these primary respondents, 472 (44%) slept in the forest, 92 (9%) slept on a farm, 132 (12%) slept in both the forest and on a farm (both sites), and 378 (35%) did not sleep in either the forest or on a farm (slept at home) within the last 12 months.

### Demographics

Of the primary respondents, 865 (81%) were male, with a median age of 40 (interquartile range [IQR] 33, 49). Primary respondents’ ethnicities consisted of Kinh, Cham, Bana, Jrai, Muong, and Thai, of which a majority were Kinh and Cham, each accounting for approximately 45% (481 and 478, respectively). The majority of people identifying as Kinh or Cham ethnicity slept in the forest: 237 (49%) and 207 (43%), respectively (χ^2^ (1; 959) = 3.4, *P* = 0.064). In regards to education, 24% (261) of respondents self-identified as illiterate, 33% (357) reported a primary school level education, and 43% (456) had secondary level or above education. Individuals who self-identified as illiterate constituted about 21% (99) of respondents who slept in the forest, 46% (42) who slept on farms, 34% (45) who slept at both sites and 20% (75) who slept at home. Socio-demographic data of primary household respondents by sleeping sites are shown in Table 1.

**Table 1:** Socio-demographic characteristics of primary household respondents (heads of household) by sleeping site in Dong Xuan District, Phu Yen Province, 2016.

Characteristics	Slept on farm (n = 92)	Slept in forest (n = 472)	Slept at both sites (n = 132)	Slept at home (n = 378)	Total (n = 1074)
Age (median, interquartile range)	47 (36, 57)	38 (32, 46)	37 (30, 44)	44 (36, 54)	41 (33, 50)
Gender (n, %)					
Male	77 (83.7)	424 (89.8)	117 (88.6)	247 (65.3)	865 (80.5)
Female	15 (16.3)	48 (10.2)	15 (11.4)	131 (34.7)	209 (19.5)
Education level (n, %)					
Illiterate	42 (45.7)	99 (21.0)	45 (34.1)	75 (19.8)	261 (24.3)
Primary school	30 (32.6)	162 (24.3)	47 (35.6)	118 (31.2)	357 (33.2)
Secondary school or above	20 (21.7)	211 (44.7)	40 (30.3)	185 (48.9)	456 (42.5)
Ethnicity (n, %)					
Cham	51 (10.7)	207 (43.3)	101 (21.1)	119 (24.9)	478 (44.5)
Kinh	9 (1.9)	237 (49.3)	5 (1.0)	230 (47.8)	481 (44.8)
Others (Bana, Jrai, Muong, Thai)	32 (27.8)	28 (24.4)	26 (22.6)	29 (25.2)	115 (10.7)

Of the 3137 associated household members included in the study, the median age was 20 (IQR 11–32), of which the lowest median was in the home group (18, IQR 10–30). In general, the proportion of male household members (42%) was lower than that of females (58%). A majority of household members were the children (2001; 64%) and partners (866; 28%) of the primary respondents. Basic socio-demographic characteristics of household members by sleeping sites are shown in Table 2.

**Table 2:** Socio-demographic characteristics of respondents’ associated household members by sleeping sites in Dong Xuan District, Phu Yen Province, 2016.

Characteristics	Slept on farm (n = 163)	Slept in forest (n = 176)	Slept at both sites (n = 125)	Slept at home (n = 2673)	Total (n = 3137)
Median age (interquartile range)	31 (22–43)	26.5 (22–37)	31 (24–41)	18 (10–30)	20 (11–32)
Gender (n, %)					
Male	51 (31.3)	107 (60.8)	49 (39.2)	1106 (41.4)	1313 (41.9)
Female	112 (68.7)	69 (39.2)	76 (60.8)	1567 (58.6)	1824 (58.1)
Education levels (n, %)					
Illiterate	91 (55.8)	41 (23.3)	61 (48.8)	667 (25.0)	860 (27.4)
Primary school	32 (19.6)	59 (33.5)	32 (25.6)	739 (27.6)	862 (27.5)
Secondary school or above	40 (24.5)	76 (43.2)	32 (25.6)	1267 (47.4)	1415 (45.1)
Ethnicity (n, %)					
Cham	116 (71.2)	89 (50.6)	86 (68.8)	1177 (44.0)	1468 (46.8)
Kinh	5 (3.1)	73 (41.5)	6 (4.8)	1244 (46.5)	1328 (42.3)
Others (Bana, Jrai, Muong, Thai)	42 (25.8)	14 (8.0)	33 (26.4)	252 (9.4)	341 (10.9)
Household relationship (n, %)					
Householder (HH)	7 (4.3)	13 (7.4)	11 (8.8)	71 (2.7)	102 (3.3)
HH’s partner	81 (49.7)	65 (36.9)	61 (48.8)	659 (24.7)	866 (27.6)
HH’s child	73 (44.8)	96 (54.5)	52 (41.6)	1780 (66.6)	2001 (63.8)
HH’s grandchild	0 (0.0)	2 (1.1)	1 (0.8)	154 (5.8)	157 (5.0)
HH’s parents	2 (1.2)	0 (0.0)	0 (0.0)	9 (0.3)	11 (0.4)

### Net use, sleeping characteristics at forest and farm sleeping sites, and occupation

Net usage of primary respondents who reported to sleep either only in the forest (472) or only on a farm (92) when working away from their village-based household residence was also captured. Overall, most respondents (400; 71%) did not sleep under nets either in the forest or on a farm. The proportion of those who slept under treated nets and untreated nets was 11% (64) and 18% (100), respectively. The odds of sleeping under treated nets in the forest was 0.34 (95% CI 0.19, 0.60) times the odds of sleeping under treated nets on a farm, but decreased after adjusting for ethnicity (aPOR = 0.30; 95% CI 0.15, 0.64), age (aPOR = 0.28; 95% CI 0.15, 0.55), and education (aPOR = 0.28; 95% CI 0.15, 0.55). The likelihood of not using nets was not significantly different for forest and farm groups (POR = 1.22; 95% CI 0.76, 1.97) (Table 3).

**Table 3:** Characteristics of primary respondents’ activities by sleeping sites in Dong Xuan District, Phu Yen Province, 2016.

Characteristics	Slept in forest (n = 472) (n, %)	Slept on farm (n = 92) (n, %)	Total (n = 564) (n, %)	POR (95% CI)	aPOR (95% CI)
Use of nets					
Treated nets	43 (9.1)	21 (22.8)	64 (11.3)	0.34 (0.19, 0.60)	0.30 (0.15, 0.64) (*ethnicity*); 0.28 (0.15, 0.55) (*education*); 0.28 (0.15, 0.55) (*age*)
Untreated nets	91 (19.3)	9 (9.8)	100 (17.7)	2.20 (1.08,4.48)	
Not use nets	338 (71.6)	62 (67.4)	400 (70.9)	1.22 (0.76, 1.97)	
Trips per year					
10 trips or less	310 (65.7)	50 (54.3)	360 (63.8)	1.61 (1.03, 2.52)	
> 10 trips or more	162 (34.3)	42 (45.7)	204 (36.2)		
Nights per trip					
7 nights or less	282 (59.7)	76 (82.6)	358 (63.5)	0.31 (0.18, 0.55)	
> 7 nights	190 (40.3)	16 (17.4)	206 (36.5)		
Type of hut					
Hut with wall	11 (2.3)	18 (19.6)	29 (5.1)	0.10 (0.05, 0.21)0	0.14 (0.05, 0.29) (*age*); 0.07 (0.03, 0.18) (*education*) 0.084 (0.034, 0.22) (*age and ethnicity*: *Cham*)
Hut without wall	460 (97.5)	73 (79.3)	533 (94.5)	9.19 (4.40, 19.19)	7.01 (3.39, 18.84) (*age*); 13.23 (5.29, 31.38) (*education*); 8.28 (3.59, 20.88) (*age and ethnicity*: *Cham*)
Slept outside	1 (0.2)	1 (1.1)	2 (0.3)		
Main work					
Exploitation (planting, logging, hunting, etc.)	453 (96.0)	6 (6.5)	459 (81.4)	341.74 (136.60, 854.94)	309.77 (110.30, 919.58) (*education*)
Farming	7 (1.5)	86 (93.5)	98 (17.4)		
Other work	12 (2.5)	0 (0.0)	7 (1.2)		
Work for					
Family	353 (74.8)	92 (100)	445 (78.9)		
Company	114 (24.2)	0 (0.0)	114 (20.2)		
Others	5 (1.1)	0 (0.0)	5 (0.9)		
Secondary work					
Exploitation	73 (15.5)	3 (3.3)	76 (13.5)	5.43 (1.81, 16.25)	
Farming	39 (8.3)	39 (42.4)	78 (3.8)	0.12 (0.07, 0.21)	0.23 (0.12, 0.40) (*ethnicity*)
No other work	360 (76.3)	50 (54.3)	410 (72.7)	2.70 (1.71, 4.28)	1.71 (1.00, 2.95) (*ethnicity*)
Work at night					
Sometimes	23 (4.9)	4 (4.3)	27 (4.8)	1.13 (0.40, 3.17)	
Never	449 (95.1)	88 (95.7)	537 (95.2)		

a, adjusted; POR, prevalence odds ratio; CI, confidence interval.

A majority of primary respondents (360; 64%) reported ≤10 trips to a forest/farm site per year and were more likely to go to the forest than a farm (POR = 1.61; 95% CI 1.03, 2.52). Analysis of these respondents who stayed seven nights or less per trip in forests or on farms (358; 64%) revealed forest workers were less likely to stay ≤7 nights than farm workers (POR = 0.31; 95% CI 0.18, 0.55). Nearly all respondents (533; 94%) reporting sleeping in huts without walls, however there was the proportion of respondents who slept in a hut without walls was significantly higher in the forest goers (POR = 9.19; 95% CI 4.40, 19.19). A younger age (< 40 years) and lower level of education were significant risk factors for staying in huts without walls (aPOR = 7.01; 95% CI 3.39, 18.84 and aPOR = 13.23; 95% CI 5.29, 31.38); age remained significant after inclusion of ethnicity (aPOR = 8.28; 95% CI 3.59, 20.88).

The main occupation of forest-going primary respondents was tree planting, logging and hunting (96%), while the primary work of those sleeping at a farm was farming (94%). Forest workers were more likely than farm workers to not have other work (POR = 2.70 (95% CI 1.71, 4.28), but this association was marginal after adjustment for ethnicity (aPOR = 1.71; 95% CI 1.00, 2.95). Nearly all respondents (95%) stated they did not work at night, there was no significant difference between the forest and farm groups (POR = 1.13, 95% CI 0.40, 3.17). Net use and sleeping characteristics of primary respondents reported to sleep either only in the forest or on a farm when working away from their village-based household residence are displayed in Table 3.

### Differences in malaria risk factors between forest and farm sleepers

There were 301 forest and farm sleeping primary respondents who agreed to answer a detailed questionnaire relating to malaria risk factors at their respective forest or farm sleeping sites. These data indicated a significantly higher proportion of huts in the forest that slept more than two people compared to huts on a farm (POR = 3.65, 95% CI 1.86, 7.16). The likelihood became more pronounced after adjustment for ethnicity (aPOR = 4.34, 95% CI 1.79, 10.68), but slightly attenuated after adjustment for education (aPOR = 3.29, 95% CI 1.60, 7.57). Of respondents who reported sleeping in the forest, 69% (179) took more than 60 minutes to reach their huts in the forest by motorbike, while 79% (34) of farm-dwelling respondents reached their huts within 60 minutes or less by the same means of transport (POR = 8.56, 95% CI 3.98, 18.40), this difference was reduced after adjusting for ethnicity (aPOR = 6.55; 95% CI 2.75, 17.28). The duration of additional walking time after riding a motorbike was similar for those who slept in forests and on farms (POR = 0.87, 95% CI 0.46, 1.65). Among the 258 respondents sleeping in the forest, 103 (40%) slept there during the rainy months (from October–December), while 65% of farm workers (28) slept on a farm during the rainy months; this difference was not significant after adjusting for ethnicity (aPOR = 1.34; 95% CI 0.59, 3.04) and education (aPOR = 2.04; 95% CI 0.95, 4.45).

The proportion of forest workers and farm workers who performed indoor activities between dusk and sleep was similar; with 200 (76%) forest workers and 33 (77%) farm workers performing indoor activities between dusk and going to sleep. Of 301 respondents, only 17 worked at night, with 16 of these working in the forest (i.e., hunting, charcoal, aloe wood, raising cattle and poultry), and one trading on a farm. Over 80% of the respondents went to sleep before 9 p.m. The difference was significant between those who slept in a forest and those who slept on a farm (POR = 0.10, 95% CI 0.02–0.52). Survey responses indicated that 64% (191) of respondents did not use a net of any type. The odds of nearly always sleeping under a net was significantly higher for respondents sleeping in the forest than for respondents sleeping on farms (POR = 2.76, 95% CI 1.14, 6.72), but was not significant after adjusting for education (aPOR = 2.46; 95% CI 0.94, 7.98). Table 4 provides a summary of risk factor data for respondents at forest or farm sleeping sites captured in the survey.

**Table 4:** Risk factors of respondents who slept in the forest versus on a farm.

Characteristics	Slept in forest (n = 258) (n, %)	Slept on farm (n = 43) (n, %)	Total (n = 301) (n, %)	POR (95% CI)	aPOR (95% CI)
Number of people sleeping in a hut					
> 2 people	212 (82.2)	24 (55.8)	236 (78.4)	3.65 (1.86, 7.16)	4.34 (1.79, 10.68) (*ethnicity*); 3.29 (1.60, 7.57) (*education*)
2 people or less	46 (17.8)	19 (44.2)	65 (21.6)		
Duration from home to hut by motorbike					
> 60 minutes	179 (69.4)	9 (20.9)	188 (62.5)	8.56 (3.98, 18.40)	6.55 (2.75, 17.28) (*ethnicity*)
60 minutes or less	79 (30.6)	34 (79.1)	113 (37.5)		
Additional time by walking					
30 minutes or more	135 (52.3)	24 (55.8)	159 (52.8)	0.87 (0.46, 1.65)	
<30 minutes	123 (47.7)	19 (44.2)	142 (47.2)		
Month slept at sleeping sites					
January-September	155 (60.1)	15 (34.9)	170 (56.5)	2.81 (1.44, 5.47)	1.34 (0.59, 3.04) (*ethnicity*); 2.04 (0.95, 4.45) (*education*)
October-December (rainy)	103 (39.9)	28 (65.1)	131 (43.5)		
Bathing time					
Before dusk (before 6 pm)	225 (87.2)	3 (7.0)	228 (75.7)	90.91 (28.77, 287.31)	
From dusk	25 (9.7)	0 (0.0)	25 (8.3)		
No bathing	8 (3.1)	40 (93.0)	48 (15.9)		
Activities from dusk to sleep					
Outdoor	58 (22.5)	10 (23.3)	68 (22.6)	0.96 (0.45, 2.03)	
Indoor	200 (77.5)	33 (76.7)	233 (77.4)		
Time go to sleep					
Before 21:00	208 (80.6)	42 (97.7)	250 (83.1)	0.10 (0.02, 0.52)	
From 21:00	50 (19.4)	1 (2.3)	51 (16.9)		
	Forest (n = 103) (n, %)	Farm (n = 43) (n, %)	Total (n = 146) (n, %)		
Frequency of net use					
Never	26 (25.2)	10 (23.3)	36 (27.4)	1.14 (0.49, 2.53)	
Sometimes	11 (10.7)	13 (30.2)	24 (16.4.0)	0.28 (0.14, 0.67)	
Regularly	30 (29.1)	13 (30.2)	43 (29.5)	0.95 (0.44, 2.04)	
Nearly always	36 (35.0)	7 (16.3)	43 (29.5)	2.76 (1.14, 6.72)	2.46 (0.94, 7.98) (*education*)

### Perceived knowledge of malaria

Survey responses indicated that of the 301 respondents who participated in the malaria risk factor component of the survey, 223 (74%) had knowledge of the causes of malaria and 229 (76%) had an understanding of the dangers of malaria. Of these respondents, 105 (35%) had some perception of malaria prevention, with 82 (78%) answering at least two of six related questions correctly, 11 (10%) correctly answering at least three questions, and 12 (11%) correctly answering at least four questions. There was no significant difference in knowledge between respondents who slept in a forest or on a farm.

Of 116 respondents who reported having malaria within the last three years, 113 slept in the forest and three slept on farms. Those with a history of malaria reported experiencing a total of 208 episodes of malaria infections collectively within three years; 204 cases occurred among respondents who slept in the forest (1.8 cases per person) and four cases occurred among respondents who slept on a farm (1.3 cases per person).

Of the respondents who slept in a forest, only 31% (79) requested standby malaria drugs from local health centers, while no respondents who slept on a farm asked for standby malaria drugs. Of the respondents who slept in a forest, 77 (30%) took malaria drugs by themselves without health consultation when they suspected malaria infections, while only one respondent (2.3%) at a farm reported this behavior. Of the 78 self-treated respondents, 61 (78%) stated they took a full dose, while 11 (14%) took a partial dose and 6 (8%) did not remember the dose they took. Table 5 provides a summary of malaria prevention knowledge, attitudes and practices.

**Table 5:** Malaria prevention knowledge, attitudes and practices among respondents who slept in the forest or on a farm.

Characteristics	Slept in forest (n = 258) (n, %)	Slept on farm (n = 43) (n, %)	Total (n = 301) (n, %)	POR (95% CI)
Know causes of malaria	188 (72.9)	35 (81.4)	223 (74.1)	0.61 (0.28, 1.36)
Know how to prevent malaria	72 (27.9)	11 (25.6)	83 (27.6)	1.13 (0.55, 2.33)
Good understanding of dangers of malaria	194 (75.2)	35 (81.4)	229 (76.1)	0.69 (0.31, 1.54)
Had malaria within last three years	113 (43.8)	3 (7.0)	116 (38.5)	10.39 (3.39, 31.82)
Carry standby malaria treatment	79 (30.6)	0 (0.0)	79 (26.2)	-
Self-treatment without health consultation	77 (29.8)	1 (2.3)	78 (25.9)	17.87 (3.43, 93.02)
Took malaria drugs (n = 78)				
Partial	11 (14.3)	0 (0.0)	11 (14.1)	
Full	60 (77.9)	1 (100)	61 (78.2)	
Did not remember	6 (7.8)	0 (0.0)	6 (7.7)	

### Key findings in relation to Vietnam malaria elimination targets

Aggregated results of key findings from the study are compiled and compared with national malaria elimination targets and described in Table 6.

**Table 6:** Comparison of key survey results to national strategy malaria indicators.

*Key Area*	Net Ownership
*National Strategy Indicators*:	By 2015, ITNs/long-lasting insecticidal nets (LLINs) ownership was 2 persons/1 double bed net in households in high and moderate malaria endemic areas, on average
*Study Findings*:	1.16 (95% CI 1.10–1.22) nets (single or double, treated or untreated) per household among all 1074 surveyed households
	1.68 (95% CI 1.63–1.74) nets (single or double, treated or untreated) per household among 740 households that reported having nets
*Key Area*:	Net Use
*National Strategy Indicators*:	By 2015, over 85% of the population at high risk of malaria (working and staying overnight in the forest) apply malaria control measures (ITNs/LLINs and other personal protection measures)
	By 2020, the proportion of high-risk populations to apply malaria control measures will exceed 95%
*Study Findings*:	164 (29%) of 564 of respondents who worked and slept in either the forest or farms used nets (treated or untreated)
	64 (11%) of 564 respondents who worked and slept in either the forest or farms used treated nets
*Key Area*:	Access to Correct Diagnosis and Treatment
*National Strategy Indicators*:	By 2015, 95% of malaria cases received correct and proper treatment in accordance with the diagnosis and treatment guidance of the Ministry of Health
	By 2020, the proportion will exceed 98%
*Study Findings*:	71 (76%) of 93 self-treated malaria patients took full course of malaria drugs (71 (46%) of total of 153 malaria patients)
	79 (17%) of 472 forest goers took standby malaria drugs when going to the forest
*Key Area*:	Knowledge of Correct Prevention Methods
*National Strategy Indicators*:	By 2015, over 95% of the population in malaria endemic areas can recall at least four key messages on malaria control and elimination
	By 2020, over 98% can recall at least four key messages on malaria control and elimination (it was 89.4% in 2009)
*Study Findings*:	12 (4%) of 301 respondents who answered questions about malaria risk factors could recall at least four key messages on malaria control and elimination

National strategic indicators sourced from the 2014 Vietnam NIMPE national malaria program final report and plan for 2015 [4].

## Discussion

This study details findings from the one of the first household level malaria indicator surveys conducted in Vietnam since the adoption of the most recent national strategy for the control and elimination of malaria [6]. Of note, the study site borders two Tier 1 Artemisinin Resistant provinces (as defined by the WHO Global plan for artemisinin resistance containment [26]), with a high risk of drug-resistant malaria, and has a significant proportion of high-risk populations, including those living and working on forest fringe farms and within the forest and ethnic minority groups. Given the study-site location, together with the targeted approach aimed at capturing data among high-risk priority populations in remote difficult-to-access settings, the results from this malaria indicator survey are significant, particularly in the context of Vietnam’s national malaria elimination strategies and associated operational priorities. Although Vietnam has undoubtedly made good progress towards its national targets, challenges such as increasingly remote transmission sites, mobile populations, and the heterogeneity of priority high-risk groups, have made it difficult to successfully increase and sustain malaria reduction in priority areas. It is clear from the results of this study that net usage, proper use of malaria treatments and the knowledge of malaria control and prevention methods among forest goers in Phu Yen Province lag behind national strategy malaria key indicators, making it difficult to meet the country’s malaria elimination goal.

As acknowledged in the Vietnam National Strategy for Malaria Control and Elimination, improving knowledge and encouraging positive behavior change among populations is central to ensuring the implementation of malaria prevention and response measures by at-risk populations [2,4,6]. Although a demonstrated understanding of the causes and dangers of malaria was high among the groups surveyed in this study, only a limited proportion of respondents from any group were able to correctly identify how to prevent malaria. This finding suggests that the higher malaria prevalence among forest-sleepers compared to those who slept on farms is likely due to different exposure risks, behaviors and prevention practices among the two groups, rather than differences in knowledge. It may also indicate a need to adjust behavior change communication (BCC) materials and strategies, given the majority of study participants were aware of the cause of malaria, but were unsure about how to prevent it. Lack of knowledge about malaria prevention measures is further demonstrated by the low use of treated nets despite national efforts to ensure net distribution among all individuals at risk of malaria [27–29].

Results of this study confirm the frequency of known high risk activity within these priority populations, such as working and sleeping in the forest or on forest fringe farms [30–32]. These results also highlight the different levels of risk and malaria prevention practices among these groups. As noted in previous research, MMPs are not homogenous groups—different structural types of human mobility show differential risk and vulnerability, and it is important to consider differences among MMPs including characteristics of movements, variances in activities and occupations, the behavior of populations, and ethnicity and cultural practices [31]. Similarly, these study results highlight how variances related to remote-area sleeping site location should also be considered when designing and targeting appropriate, foci-specific strategies to support malaria elimination in these settings. For example, significant differences reflected in the study results between respondents who slept in forests versus on farms to be considered when developing malaria prevention and elimination policies include: (i) respondents who slept in the forest were more likely to sleep in a hut without walls and use untreated nets compared to those sleeping on farms, suggesting different exposure to mosquitos and opportunities to tailor prevention strategies; (ii) a higher proportion of individuals sleeping in forests slept in huts with more than two people suggest that further case follow-up and investigation of forest goers could yield additional cases; and (iii) forest sleepers generally travelled greater distances, with a higher proportion having to travel more than 60 minutes by motorbike from their home, indicating that suspected forest transmission source locations may be difficult to access, and targeting these sites will likely be resource intensive and time consuming.

While previous research within the Greater Mekong Subregion has demonstrated the positive impacts of long-lasting insecticide-treated hammock nets (LLHNs) against forest malaria vectors [33–35], this study indicates very low mosquito net usage (either treated or untreated) among those sleeping in the forest and on farms. An additional important finding was that most individuals used some form of shelter when sleeping remotely, however these shelters often lacked walls. This may provide an opportunity to explore novel screening or protective strategies that may differ from regular bed net or hammock options. Of note, most respondents said they rarely worked at night, suggesting that improved hut construction, increased use of insecticide treated mosquito or hammock nets, or adapted shelter screening may reduce some exposure to malaria vectors, at the very least those with a tendency to feed at dusk and throughout the night. Additional personal protective measures such as topical or spatial repellents and insecticide treated clothing could also be considered for individuals sleeping in forest settings, although further research is still required in regards to their level of protective effects against malaria [36]

There were significant proportions of self-identified illiterate individuals and ethnic minorities among the respondents. More detailed ethnographic data are required to identify the reasons for the high levels of illiteracy and the discrepancies in knowledge about malaria prevention. Additionally, exploration of the relationship between gender, literacy, level of exposure and infection would be of interest for the development of new targeted interventions. Such studies would be especially useful in determining why those with adequate knowledge do not adopt more appropriate health behaviors and would enable the creation of appropriate malaria-based BCC activities and campaigns, in the context of both the educational levels and cultural practices.

In certain high-risk population groups in Vietnam, the use of standby malaria drugs for self-medication is included within the national treatment guidelines [37]. The survey highlights a number of forest sleepers that used standby malaria drugs, with a high proportion reporting that when these were taken, they completed a full course of the medication. As these results were based on self-reporting, a risk exists of respondents providing perceived socially desirable responses. However, even if the high rate of treatment completion is correct, a significant number of complexities exist in regards to this self-regulated practice including how soon treatment was initiated after becoming symptomatic, the likelihood of actually being infected with malaria, dosing regimens, and the known presence of asymptomatic malaria infections. Asymptomatic malaria infections can outnumber clinical cases [38] and despite very low parasite densities, are still important reservoirs for malaria infection transmission [39]. Standby treatment could be explored as a more widely used intervention for individuals in these high-risk areas. However, the lack of oversight by health professionals can lead to incomplete or inappropriate treatment, particularly as this approach is not based on confirmed parasitological diagnosis. Better understanding of this behavior could inform future malaria prevention and treatment policies for high priority populations.

In line with national objectives to strengthen surveillance capacity in these high priority regions [6,19], more routine information is needed regarding additional individuals sharing these remote-area sleeping sites to better define the at-risk populations. In particular, it is important to ascertain where these individuals travel from and where they travel to after leaving the forests or farms. By doing so, potential opportunities for implementing focused measures such as active case detection and screening of MMPs upon return to their village residence could be explored. The mobile and migrant nature of many people in these areas is also a major factor in the continued re-introduction of malaria into these zones. The inclusion of blood sampling to genetically characterize parasites in these areas may assist in determining the geographic transmission pathways of these parasites (i.e. matching genotypes of parasites in these areas to the genotypes of parasites from other areas). Genotyping these parasites should also include assessing the profile of antimalarial susceptibility to ensure adequate drugs are being deployed. Given the nature of the remote locations however, implementing such activities has obvious logistical constraints.

Findings from this study suggest it is likely that transmission is occurring in forest and forest fringe settings that vary in distance and travel time from people’s fixed village homes. Such a finding provides an opportunity for the exploration of using mobile/migrant malaria workers (MMWs) at these sites, similar to the way such MMWs operate in villages and on farms in Cambodia [40]. Routinely visiting suspected transmission sites, capturing relevant up-to-date data, and engaging with MMWs to implement malaria prevention activities such as awareness education, BCC, sleeping site assessments, provision of vector control measures, and testing and treatment, may present an effective approach to supporting elimination in these mobile populations that may not be best served by traditional residential village-based prevention activities. Given the geographic context of these settings, the adoption of geospatial surveillance systems could provide a platform moving forward for better targeted foci-specific interventions to remote-area transmission sites [18,25]

The malaria indicator survey conducted in this study, while informative, included some limitations. A reliance on self-reported data that could not be verified through observation of behaviors or confirmation of reported cases using blood tests introduced potential bias. While efforts were made to ensure local-level health staff well established within their respective communities led the interview process, and emphasis was placed on the voluntary and confidential nature of the survey, potential limitations associated with participant reporting and interviewer positionality may be considered. Additionally, this survey was also limited in size and scope, focusing on only one of the many high-priority regions of Vietnam. Having been the first detailed survey conducted since adoption of the current national elimination strategy, results from this study strongly support a need to implement more formalized and targeted population-at-risk malaria indicator surveys throughout Vietnam to monitor and assess progress against priority elimination objectives, particularly given the diversity of ethnicities living and working in high-risk locations throughout the country.

Results of this study have provided a detailed snapshot of the challenges facing malaria elimination in a priority area of Vietnam and highlight potential opportunities to utilize these data that were captured to target tailored intervention strategies to high-risk population groups. Targeting appropriate interventions to high-risk individuals sleeping in forests or on farms will be essential to ensuring that Vietnam achieves its elimination goals. To support this outcome, conducting routine data collection within these populations, as well as at suspected malaria transmission forest and farm sites is essential to ensure appropriate and effective location-specific interventions are implemented. At the regional level, this study highlights some of the complex challenges facing malaria elimination within the Greater Mekong Subregion, and provides insight in potential strategies to both capture essential data and characterize specific high-risk groups including mobile and migrant populations, specifically those frequenting remote forest and farm settings.

## Supporting information

S1 File Household level malaria indicator survey template used in the study. (PDF)
