# Supplementary material for: Results from a malaria indicator survey highlight the importance of routine data capture in high-risk forest and farm transmission sites in Vietnam to tailor location-specific malaria elimination interventions
Source: PLoS One. 2021 Apr 16;16(4):e0250045. doi: 10.1371/journal.pone.0250045 (PMC8051764; doi:10.1371/journal.pone.0250045)
Supplement: S1 File — (PDF) [file pone.0250045.s001.pdf]

Long_HH_05Sep15_Bang_hoi_gia_dinh_final

1. Mã số hộ, nếu không có sử dụng tên của chủ hộ
Household number if available, if not, house owner

---

2. Họ tên người trả lời (Chỉ hỏi chủ hộ là người đi rừng, nếu không hỏi người có kinh nghiệm đi rừng trong hộ)- (Viết tắt chữ cái đầu của họ đệm và tên) vd: Nguyễn Văn Anh (viết N.V.A)
Interviewee initials (HH head if FG, if not senior FG present)

---

3. Ông/bà có đi ngủ rừng hoặc ngủ rẫy gần rừng?
Is this a senior FG or FF farmer (if there is in the house) - if not most senior available person under 60; if wrong person stop and need to identify a correct person

- ○ Có
- ○ Không

4. Dân tộc
Ethnicity

- ○ Kinh
- ○ Ê-đê
- ○ Ba-na
- ○ Chăm
- ○ Vân Kiều
- ○ Tà-ôi
- ○ Pa-co
- ○ Khác

5. Năm sinh

[illegible]
Year of birth

____

6. Giới tính
Gender

- ◯ Nam
- ◯ Nữ

7. Trình độ học vấn cao nhất trong hộ gia đình là gì?
Highest level of education in the HH

- ◯ Không biết chữ
- ◯ Tiểu học
- ◯ Trung học cở sở
- ◯ Trung học phổ thông
- ◯ Trên trung học phổ thông

NHỮNG CÂU HỎI VỀ KIẾN THỨC VÀ THÁI ĐỘ

8. Ông/bà biết gì về nguyên nhân gây bệnh sốt rét? (để người trả lời tự nói nếu không nói được ý nào thì hỏi lại ý đó)
Do you know anything about the causes of malaria (spontaneous first and then ask)

- ☐ a. Muỗi sốt rét đốt.
- ☐ b. Rừng và bìa rừng là nơi có nhiều muỗi sốt rét
- ☐ c. Khi hỏi đồng ý với: Muỗi sốt rét chỉ đốt từ chập tối đến rạng sáng.
- ☐ d. Khi hỏi đồng ý với: Muỗi sốt rét đốt người thường không thấy đau, không thấy ngứa nhưng truyền bệnh rất nguy hiểm
- ☐ e. Khi hỏi đồng ý với: Sốt rét sẽ truyền cho người thân nếu không chữa trị kịp thời
- ☐ f. Khi hỏi đồng ý với: Rừng và bìa rừng là nơi có nhiều muỗi sốt rét
- ☐ g. Không biết

9. Theo ông/bà có những cách nào phòng tránh sốt rét/bảo vệ để không bị muỗi đốt?(để người trả lời tự nói nếu không nói được ý nào thì hỏi lại ý đó)?

How to prevent malaria/ not to be infected by mosquitoes ?

- [ ] a. Luôn luôn ngủ mùng/võng mùng khi ở nhà, khi đi rừng và khi đi rẫy
- [ ] b. Đi lấy nước và tắm ở sông/suối TRƯỚC khi trời sẩm tối
- [ ] c. Bôi kem chống muỗi lúc chập tối trước khi đi ngủ (cho các hoạt động vào buổi tối và ban đêm: săn bắn, uống rượu, xem tivi, hẹn hò, tắm, ăn tối,...)
- [ ] d. Nhớ bôi kem chống muỗi và giăng mùng trước khi uống rượu
- [ ] e. Mang mùng/võng mùng đi tẩm hóa chất khi được cán bộ y tế yêu cầu
- [ ] f. Khi có sốt phải đến ngay cơ sở y tế để xét nghiệm, chuẩn đoán và điều trị
- [ ] g. Khi hỏi đồng ý với: Luôn luôn ngủ mùng/võng mùng khi ở nhà, khi đi rừng và khi đi rẫy
- [ ] h. Khi hỏi đồng ý với: Đi lấy nước và tắm ở sông/suối TRƯỚC khi trời sẩm tối
- [ ] j. Khi hỏi đồng ý với: Bôi kem chống muỗi lúc chập tối trước khi đi ngủ (cho các hoạt động vào buổi tối và ban đêm: săn bắn, uống rượu, xem tivi, hẹn hò, tắm, ăn tối,...)
- [ ] k. Khi hỏi đồng ý với: Nhớ bôi kem chống muỗi và giăng mùng trước khi uống rượu
- [ ] l. Khi hỏi đồng ý với: Mang mùng/võng mùng đi tẩm hóa chất khi được cán bộ y tế yêu cầu
- [ ] m. Khi hỏi đồng ý với: Khi có sốt phải đến ngay cơ sở y tế để xét nghiệm, chuẩn đoán và điều trị
- [ ] n. Không biết

10. Theo ông/bà cảm thấy bệnh sốt rét nguy hiểm ở mức nào? (Từ 1 là Không nguy hiểm gì? Đến 5 là rất nguy hiểm/có thể gây chết người)

How dangerous is malaria? From level 1 to level 5 (very dangerous)

- ◯ Mức 1 (Không nguy hiểm)
- ◯ Mức 2
- ◯ Mức 3
- ◯ Mức 4
- ◯ Mức 5 (Rất nguy hiểm/có thể chết người)

11. Gia đình ông/bà có bao nhiêu người? (Những người ăn và ngủ ở nhà ông/bà 6 tháng trở lại đây)

How many people in your household ? (People ate and sleeped in last 6 months in this house)

____

12. Nhà ông/bà có người nhập cư (người từ nơi khác/tỉnh khác đến) ở không?

Any migrants in this house ?

- ◯ Có
- ◯ Không

13. Có bao nhiêu người mắc sốt rét trong 5 năm gần đây của gia đình ông/bà? (cả gia đình)

How many family members had malaria in last 5 years ?

____

14. Số lần mắc sốt rét trong 5 năm gần đây của gia đình ông/bà? (cả gia đình)

Total times had malaria in last 5 years

____

15. Số lần ông/bà mắc sốt rét trong 5 năm gần đây? (cá nhân, nếu không ghi số 0)

How many times have you had malaria in last 5 years ? (if interviewee has not had malaria, please write 0)

____

16. Lần mắc sốt rét gần đây nhất của ông/bà cách đây bao nhiêu THÁNG? (Nếu tháng nay ghi 0)

[illegible]

How long from your most recent malaria ? (by Month)

____

17. Ông/bà có vào rừng để làm việc không?

Have you ever go to forest to work?

- ○ Có
- ○ Không

NHÓM CÓ ĐI RỪNG

18. Ông/bà có ngủ ở trong rừng không? (Nếu không bỏ qua các câu liên quan ngủ trong mùng trong rừng)

Do you sleep in the forest?

- ○ Có
- ○ Không

» NHÓM CÓ NGỦ LẠI trong rừng ít nhất là 1 đêm trong 1 năm qua

19. Công việc chính (nghề chiếm nhiều thời gian nhất) của ông/bà trong rừng là gì?
What is your main work in the forest ?

- ◯ Trồng cây
- ◯ Làm than củi
- ◯ Khai thác gỗ
- ◯ Tìm trầm
- ◯ Thợ săn
- ◯ Bẫy thú
- ◯ Làm rẫy
- ◯ Công nhân
- ◯ Thợ hồ
- ◯ Khai thác quặng
- ◯ Buôn bán
- ◯ Việc khác

20. Mô tả chi tiết công việc chính của ông/bà? Làm rõ những công việc cụ thể khi làm việc đó là gì? (VD: Trồng cây gì?, săn con gì? Bẫy con gì?Loại gỗ khai thác là gì)
Most important question on the list this end the multiple choice to determine future malaria tranmission risk classification to who to give prophylaxis and hammock nets; e.g. exact type of planation or wood exploitation or hunter or trapper - improve list with local people)

____

21. Ông/bà có làm việc sau khi trời tối hoặc trước khi trời sáng?
Do you work after dark or before dawn?

- ◯ Có
- ◯ Không

22. Tên công ty/tên chủ đang làm việc?
Company/Ower Name

____

23. Số người làm tại nơi đó?
Numberworkersat_site

____

24. Ở trong rừng_Tính trung bình, thời gian Ông/bà ngủ trong chòi/lều này khoảng bao ngày trong một năm? (Ngày)
In average, how many days you sleep in forest per year ?

____

25. Loại rừng Ông/bà đã đi vào? (Chọn 1 hoặc cả hai)
Type of forest you go to? (Choices one or both)

- [ ] Rừng già/rừng sâu/Rừng nguyên sinh
- [ ] Bìa rừng (Rừng tái sinh/Rừng trồng)

26. Nhóm của ông/bà ngủ nơi đâu?
Where your work group sleep ?

- [ ] Nhà
- [ ] Lều có tường che chắn có thể phun thuốc
- [ ] Lều bạt
- [ ] Võng có mùng
- [ ] Võng không mùng
- [ ] Trên mặt đất
- [ ] Khác

27. Tháng nào thì ông/bà vào rừng làm việc này?
Select which months you spend sleeping in the forest with this work

- [ ] Tháng 1
- [ ] Tháng 2
- [ ] Tháng 3
- [ ] Tháng 4
- [ ] Tháng 5
- [ ] Tháng 6
- [ ] Tháng 7
- [ ] Tháng 8
- [ ] Tháng 9
- [ ] Tháng 10
- [ ] Tháng 11
- [ ] Tháng 12

28. Khi làm việc ở trong rừng, nơi ngủ của ông/bà có bao nhiêu người NGỦ lại ít nhất là 1 đêm trong năm qua (có thể làm thuê)? (tính luôn người được hỏi)
How many people sleep with you at the working location (may be hired labour) (include interviewee)

____

29. Trong những người ngủ này, có bao nhiều người mắc sốt rét trong vòng 2 năm trở lại đây? (tính luôn người được hỏi)
In those people sleeping with interviwee, how many people get malaria in recent 2 years (include interviewee)

____

[illegible]

30. Ông/bà biết bao nhiêu người/ca mắc sốt rét trong vùng trong vòng 2 năm qua?
How many malaria cases do you aware of in the area around in the last 2 yrs?

____

31. Có KHOẢNG bao nhiêu người ngủ trong bán kính 2km xung quang nơi làm việc của ông/bà ở trong rừng trong 2 năm trở lại đây?
How many people sleep forest in the 2km area in 2yrs? (APPROXIMATELY)

____

32. Từ chỗ ngủ trong rừng tới chỗ có sóng điện thoại ĐI BỘ mất KHOẢNG bao nhiêu PHÚT - Ghi bằng "0" nếu có sóng?
Howmany minutestonearestcellphone_coverage by walking from sleeping area (record "0" if there is signal) (APPROXIMATELY)

____

33. Khi ngủ ở RỪNG thường mấy giờ ông/bà ngủ?
What time do you sleep at FOREST?

--:-- --

34. Ông/bà có dùng mùng hay võng khi ngủ rừng không?
Do you use net/hammock when sleep?

- ◯ Có
- ◯ Không

» » NHÓM ĐI RỪNG có NGỦ MÙNG/VÕNG MÙNG

35. Nếu đo lường mức độ dùng mùng/võng CẢ ĐÊM thì nhóm của ông/bà dùng ở mức độ nào? (đọc đáp án)
How often do members of your sleepinggroup sleep under net/hammock net in the forest?

- ◯ Không bao giờ
- ◯ Thỉnh thoảng
- ◯ Thường xuyên
- ◯ Luôn luôn

36. Có tổng cộng bao nhiêu mùng và võng tại nơi ngủ của nhóm của ông/bà?
Total net and hammock do sleeping group have in sleeping area in forest

____

37. Loại mùng và võng ở đó là gì? (CHỌN TẤT CẢ CÁC LOẠI MÀ HỌ CÓ)
Types of nets and hammocks? (Select all of them)

- [ ] Mùng đơn
- [ ] Mùng đôi
- [ ] Mùng đơn tẩm thuốc MỀM
- [ ] Mùng đơn tẩm thuốc CỨNG
- [ ] Mùng đôi tẩm thuốc MỀM
- [ ] Mùng đôi tẩm thuốc CỨNG
- [ ] Mùng đơn tẩm thuốc tồn lưu MỀM
- [ ] Mùng đơn tẩm thuốc tồn lưu CỨNG
- [ ] Mùng đôi tẩm thuốc tồn lưu MỀM
- [ ] Mùng đôi tẩm thuốc tồn lưu CỨNG
- [ ] Võng không có mùng
- [ ] Võng có mùng không có khóa kéo
- [ ] Võng có mùng có khóa kéo
- [ ] Võng có mùng tẩm không có khóa kéo
- [ ] Võng có mùng tẩm có khóa kéo
- [ ] Võng có mùng tẩm tồn lưu không khóa kéo
- [ ] Võng có mùng tẩm tồn lưu có khóa kéo

- ☐ Võng không có mùng
- ☐ Võng có mùng không có khóa kéo
- ☐ Võng có mùng có khóa kéo
- ☐ Võng có mùng tẩm không có khóa kéo
- ☐ Võng có mùng tẩm có khóa kéo
- ☐ Võng có mùng tẩm tồn lưu không khóa kéo
- ☐ Võng có mùng tẩm tồn lưu có khóa kéo
- ☐ Khác

38. Nhóm của ông/bà có TỔNG CỘNG bao nhiêu MÙNG loại thường ở nơi ngủ trong rừng?
How many total regular nets for sleeping group do THEY have?

____

39. Trong đó, Có bao nhiêu cái MÙNG được tẩm hóa chất?
How many treated nets for sleeping group?

____

40. Có tổng cộng bao nhiêu cái võng tại nơi ngủ của nhóm ông/bà?
How many hammocks for sleeping group do they have?

____

41. Trong đó có bao nhiêu cái võng có mùng ở trong nhóm làm việc của ông/bà?
How many hammocks with NETs does sleeping group have?

____

42. Trong đó, có bao nhiêu cái võng có mùng được tẩm hóa chất?
How many treated hammocks do you have in sleeping group?

____

43. Ông/bà có muốn dùng mùng/võng mùng được tẩm CẢ ĐÊM khi ngủ ở trong rừng?
Do you want to use treated net/hammock when sleeping in the forest ?

- ○ Có

- ◯ Không

44. Loại mùng/võng nhóm ông/bà thích dùng NHẤT để ngủ - CẢ ĐÊM trong rừng là gì? (Cho ví dụ ông bà

Type of net/hammock your workgroup like to use the most? Show examples that we have purchased, plus program regular net and hammock

- ◯ Mùng đơn tẩm thuốc MỀM
- ◯ Mùng đơn tẩm thuốc CỨNG
- ◯ Mùng đôi tẩm thuốc MỀM
- ◯ Mùng đôi tẩm thuốc CỨNG
- ◯ Mùng đơn tẩm thuốc tồn lưu MỀM
- ◯ Mùng đơn tẩm thuốc tồn lưu CỨNG
- ◯ Mùng đôi tẩm thuốc tồn lưu MỀM
- ◯ Mùng đôi tẩm thuốc tồn lưu CỨNG
- ◯ Võng có mùng tẩm không có khóa kéo
- ◯ Võng có mùng tẩm có khóa kéo
- ◯ Võng có mùng tẩm tồn lưu không khóa kéo
- ◯ Võng có mùng tẩm tồn lưu có khóa kéo
- ◯ Võng có mùng tẩm không có khóa kéo
- ◯ Võng có mùng tẩm có khóa kéo
- ◯ Võng có mùng tẩm tồn lưu không khóa kéo
- ◯ Võng có mùng tẩm tồn lưu có khóa kéo
- ◯ [illegible]

Khác

45. Mô tả mùng/võng đó để biết chính xác nó như thế nào?

Describle net/hammock your workgroup like the most

46. Dự án muốn tìm phương án để tất cả những người đi rừng có mùng tẩm thuốc. Vậy, gia đình ông/bà muốn dự án chia sẻ bao nhiêu tiền cho việc mua mùng/võng tầm thuốc?

We want to find a way for all forest goers to have a treated net. One way is to get more expensive nets and cost-share. How much money do you want us to share to buy hammock/net?

47. Một cách khác là sử dụng mùng có giá cả rẻ hơn. Vậy loại mùng nào sau đây ông/ bà có thể chấp nhận sử dụng?
Another way would be to provide less expensive nets. What other types can be acceptable that you would still want to use?

- ☐ Mùng đơn tẩm thuốc MỀM
- ☐ Mùng đơn tẩm thuốc CỨNG
- ☐ Mùng đôi tẩm thuốc MỀM
- ☐ Mùng đôi tẩm thuốc CỨNG
- ☐ Mùng đơn tẩm thuốc tồn lưu MỀM
- ☐ Mùng đơn tẩm thuốc tồn lưu CỨNG
- ☐ Mùng đôi tẩm thuốc tồn lưu MỀM
- ☐ Mùng đôi tẩm thuốc tồn lưu CỨNG
- ☐ Võng có mùng tẩm không có khóa kéo
- ☐ Võng có mùng tẩm có khóa kéo
- ☐ Võng có mùng tẩm tồn lưu không khóa kéo
- ☐ Võng có mùng tẩm tồn lưu có khóa kéo
- ☐ Võng có mùng tẩm không có khóa kéo
- ☐ Võng có mùng tẩm có khóa kéo
- ☐ Võng có mùng tẩm tồn lưu không khóa kéo
- ☐ Võng có mùng tẩm tồn lưu có khóa kéo
- ☐ Khác

48. Theo ông/bà, giải pháp phù hợp nhất mọi người sẽ sử dụng là gì?
In the interviewers opinion, what is the most affordable solution that people will use?

---

49. Ông/bà có thích loại mùng tồn lưu cứng của Chương trình Quốc gia cấp không? (giải pháp rẻ nhất là khoảng 85.000đ)
Would the double regular nets provided by National Program be an accptable solution? (cheapest solution $3.75)

- ○ Có
- ○ Không

50. Nếu như không thích, ông bà cho biết lý do tại sao?
If no, why not?

---

51. Ông/bà có thích loại võng có mùng mà chương trình quốc gia cấp?
Would the hammock nets provided by National Program be an acceptable solution?

- ○ Có
- ○ Không

52. Nếu như không thích, ông bà cho biết lý do tại sao?
If no, why not?

---

53. Ông/bà có loại mùng/võng nào dưới đây Ở NHÀ? Đọc đáp án
Do you have net and/or hammock at home? Reading choices

- ☐ Mùng
- ☐ Võng có mùng
- ☐ Võng không có mùng nhưng có mùng kèm theo hoặc bọc võng
- ☐ Không có cả ba

54. Ông/bà có thể mang những mùng/võng có mùng này vào rừng không?
Can you bring nets/hamock that you have from home to forest?

- ○ Có
- ○ Không

55. . Nếu không mang Tại sao không?
If no, why not - consider list

- [ ] Không có do không tiền mua
- [ ] Không có do không biết chỗ mua
- [ ] Không có do xa quá không đi được để mua
- [ ] Nhà đang dùng không thể lấy đi
- [ ] Quá nặng để mang đi
- [ ] Khác

56. Ông/bà có muốn tẩm hóa chất cho mùng của ông/bà (chưa được tẩm) không?
Would you be willing to treat your nets?

- ○ Có
- ○ Không

57. Ông/bà có muốn tẩm hóa chất cho cả võng và mùng của võng ông/bà (chưa được tẩm) không?
Would you be willing to treat your entire nets/hammock ?

- ○ Có
- ○ Không

58. Nếu không tẩm cả võng và mùng của võng, thì chỉ tẩm mùng của võng được không?
If no, Would you be willing to treat just only the net of hammocks?

- ○ Có
- ○ Không

59. Theo đánh giá của cán bộ điều tra, có bao nhiêu màn cần được tẩm cho nhóm ngủ trong rừng này ?
In the interviewer's judgement, how many nets need to be treated for this work group?

60. Theo đánh giá của cán bộ điều tra, cần mua bao nhiêu mùng mới cho nhóm ngủ trong rừng này?
In the interviewer's judgement, how many nets need to be purchased?

____

61. Theo đánh giá của cán bộ điều tra, loại nào sau đây cần được mua mới?
In the interviewer's judgement, which types need to be purchased?

- [ ] Mùng đơn tẩm thuốc MỀM
- [ ] Mùng đơn tẩm thuốc CỨNG
- [ ] Mùng đôi tẩm thuốc MỀM
- [ ] Mùng đôi tẩm thuốc CỨNG
- [ ] Mùng đơn tẩm thuốc tồn lưu MỀM
- [ ] Mùng đơn tẩm thuốc tồn lưu CỨNG
- [ ] Mùng đôi tẩm thuốc tồn lưu MỀM
- [ ] Mùng đôi tẩm thuốc tồn lưu CỨNG
- [ ] Võng có mùng tẩm không có khóa kéo
- [ ] Võng có mùng tẩm có khóa kéo
- [ ] Võng có mùng tẩm tồn lưu không khóa kéo
- [ ] Võng có mùng tẩm tồn lưu có khóa kéo
- [ ] Võng có mùng tẩm không có khóa kéo
- [ ] Võng có mùng tẩm có khóa kéo
- [ ] Võng có mùng tẩm tồn lưu không khóa kéo
- [ ] Võng có mùng tẩm tồn lưu có khóa kéo
- [ ] Khác

62. Theo đánh giá của cán bộ điều tra, loại màn nào có giá cả hợp lý nhất và được chấp nhận nhiều nhất (số lượng và loại) cho nhóm ngủ trong rừng này? (Bao nhiêu cái và loại nào? Thêm giá vào mỗi cái mùng)

In the interviewer's judgement, what is the most affordable, acceptable solution for this sleeping group?(Number of nets and which type)? (Add cost of each net to the list)

____

63. Theo đánh giá của cán bộ điều tra, giải pháp nào được cho là hợp lý nhất để giảm trừ lây truyền sốt rét cho nhóm ngủ trong rừng này? (số mùng và loại mùng mà nhóm này thực sự sẽ dùng)

In the interviewer's judgement, what is the best solution to reduce malaria transmission for this sleeping group (Number of nets and which type that people will really use)?

____

64. Theo đánh giá của điều tra viên, Nếu loại màn cần mua có giá cao, nhóm ngủ trong rừng này có sẵn sàng chi trả 1/2 giá tiền với dự án không? (có thể hỏi người dân để trả lời câu hỏi này)

In the interviewer's judgement, If the chosen type of net has high price, will the people in this working group be willing and able to share half cost with project?

- ◯ Có
- ◯ Không

65. Ở trong rừng, lúc chập tối đến rạng sáng, ông/bà thường bị muỗi đốt khi đang làm gì - kể tất cả?

What do you do when mosquitoes bother you? (Select all)

- ☐ Đi tắm
- ☐ Ăn cơm
- ☐ Nấu cơm
- ☐ Đang làm việc
- ☐ Đi săn
- ☐ Ngồi nghỉ
- ☐ Ngủ
- ☐ Muỗi không chích tôi

66. Ở trong rừng_Làm gì thì bị muỗi cắn nhiều nhất
What do you do at the time when mosquitoes bother you most - Main

- ◯ Đi tắm
- ◯ Ăn cơm
- ◯ Nấu cơm
- ◯ Đang làm việc
- ◯ Đi săn
- ◯ Ngồi nghỉ
- ◯ Ngủ
- ◯ Muỗi không chích tôi

67. Ông/bà có muốn dùng kem bôi chống muỗi không?
Would you be willing to use mosquito repellant ?

- ◯ Có
- ◯ Không

68. Ông/bà có muốn uống thuốc phòng sốt rét trước khi đi vào rừng không? (UỐNG MỖI VIÊN MỘT NGÀY SAU KHI ĂN LÚC Ở RỪNG)
Would you be willing to take medicine before you go to the forest in the future to prevent malaria? (one pill per day with meals while in forest)

- ◯ Có
- ◯ Không

69. Ở trong rừng, Ông/bà có đi lấy nước sinh hoạt/đi ra ngoài sau khi trời tối hoặc trước khi trời sáng không?
Is patient going to get drinking water outside location after dark or before dawn?

- ◯ Có
- ◯ Không

70. Ở trong rừng, Ông/bà có đi tắm ở suối/khe nước sau khi trời tối không?
Bathing outside house after dark

- ◯ Có
- ◯ Không

71. Công việc thứ hai của ông/bà tại rừng là gì? (Nếu có) (Nếu không có chuyển qua câu 76)
Other types of work in the forest (If no, move to question 76)

- ☐ Trồng cây
- ☐ Làm than củi
- ☐ Khai thác gỗ
- ☐ Tìm trầm
- ☐ Thợ săn
- ☐ Bẫy thú
- ☐ Làm rẫy
- ☐ Công nhân
- ☐ Thợ hồ
- ☐ Khai thác quặng
- ☐ Buôn bán
- ☐ Việc khác
- ☐ Không có

72. Mô tả chi tiết công việc thứ hai của ông/bà? Làm rõ những công việc cụ thể khi làm việc đó là gì?
Most important question on the list this end the multiple choice to determine future malaria tranmission risk classification to who to give prophylaxis and hammock nets: e.g. exact type of planation or wood exploitation or hunter or trapper - improve list with local people)

[illegible]

---

73. Ông/bà có làm việc sau khi trời tối hoặc trước khi trời sáng?
Do you work after dark or before dawn?

- ○ Có
- ○ Không

74. Tên công ty /tên chủ thứ 2 đang làm việc?
Company/Ower Name

---

75. Số người làm tại nơi đó?
Numberworkersat_site

---

76. Ông/bà có đi vào rẫy gần rừng không?
Do you go to farm near the forest (answer as above)

- ○ Có
- ○ Không

NHÓM CÓ ĐI RẪY GẦN RỪNG

77. Ở trong rẫy Ông/bà có ngủ ở trong chòi/lều trên rẫy không - ít nhất là 1 đêm trong 1 năm qua?
Did you sleep in the farm near forest fringe - at least 1 night in last year?

- ○ Có
- ○ Không

» NHÓM CÓ NGỦ LẠI TẠI RẪY GẦN RỪNG

78. Trong rừng, ông/bà trồng cây gì/nuôi con gì (việc chính)?
Main work when you go to farm

- ◯ Trồng cây
- ◯ Làm than củi
- ◯ Khai thác gỗ
- ◯ Tìm trầm
- ◯ Thợ săn
- ◯ Bẫy thú
- ◯ Làm rẫy
- ◯ Công nhân
- ◯ Thợ hồ
- ◯ Khai thác quặng
- ◯ Buôn bán
- ◯ Việc khác

79. Mô tả chi tiết công việc thứ hai của ông/bà? Làm rõ những công việc cụ thể khi làm việc đó là gì?
Most important question on the list this end the multiple choice to determine future malaria tranmission risk classification to who to give prophylaxis and hammock nets; e.g. exact type of planation or wood exploitation or hunter or trapper - improve list with local people)

____

80. Số người làm tại nơi đó?
Numberworkersat_farm

____

81. Ở trong RẪY GẦN RỪNG_Tính trung bình, thời gian Ông/bà ngủ trong chòi/lều này khoảng bao ngày trong một năm? (Ngày)

(Ngày)

In average, how many days you sleep in farm per year ?

____

82. Nhóm của ông/bà ngủ nơi đâu?

Where are your sleepinggroup sleep?

- ☐ Nhà
- ☐ Lều có tường che chắn có thể phun thuốc
- ☐ Lều bạt
- ☐ Võng có mùng
- ☐ Võng không mùng
- ☐ Trên mặt đất
- ☐ Khác

83. Tháng nào thì ông/bà vào RẪY làm việc này?

Select which months per year you spend sleeping in the FF with this work

- ☐ Tháng 1
- ☐ Tháng 2
- ☐ Tháng 3
- ☐ Tháng 4
- ☐ Tháng 5
- ☐ Tháng 6
- ☐ Tháng 7
- ☐ Tháng 8
- ☐ Tháng 9
- ☐ Tháng 10
- ☐ Tháng 11
- ☐ Tháng 12

84. Khi làm việc ở trong RẪY, nơi ngủ của ông/bà có bao nhiêu người NGỦ lại ít nhất là 1 đêm trong năm qua (có thể làm thuê)? (Bao gồm cả người được hỏi)

How many people sleep with you at the sleeping-working location (denominator for nets)? (Include interviewee)

____

85. Trong những người ngủ này, có bao nhiều người mắc sốt rét trong vòng 2 năm trở lại đây? (Tính luôn người được hỏi)

In those people sleeping with interviwee, how many people get malaria 2 years (include interviewee)

____

86. [illegible]

86. Tại nơi làm việc của ông/bà ở trong rẫy, có KHOẢNG bao nhiêu người ngủ trong bán kính 0,5 km trong 2 năm trở lại đây ?

HowmanypeoplesleepinhalfKm in farm in 2 yrs (APPROXIMATELY)

____

87. Trong 2 năm qua, có KHOẢNG bao nhiêu người ông bà biết bị mắc sốt rét trong phạm vi 0.5 Km tại nơi làm việc ở trong rẫy?

Howmanymalariacases do you know in2yr_farm in half km (APPROXIMATELY)

____

88. Từ chỗ ngủ trong rẫy tới chỗ có sóng điện thoại ĐI BỘ mất bao nhiêu phút - Ghi bằng "0" nếu có sóng?

Howmany minutestonearestcellphone_coverage by walking (record "0" if there is signal)

____

89. Khi ngủ ở rẫy thường mấy giờ ông/bà ngủ?

what time do you sleep at farm?

--:-- --

____

90. Ông/bà có dùng mùng hay võng khi ngủ rẫy không?

Did you use net/hammock when sleep in FF?

- ◯ Có
- ◯ Không

» » NHÓM ĐI RẪY CÓ NGỦ MÙNG/VÕNG MÙNG

91. Khi ở RẪY_nhóm của ông/bà có dùng mùng/võng HẾT CẢ ĐÊM ở mức nào? (đọc đáp án)

How often do members of your sleepinggroup under net/hammock net in the FF?

- ◯ Không bao giờ
- ◯ Thỉnh thoảng
- ◯ Thường xuyên
- ◯ Luôn luôn

92. Có tổng cộng bao nhiêu mùng và võng tại nơi ngủ của nhóm của ông/bà?

Total nets and hammocks do sleepinggroup have in sleeping in forest

____

93. Loại mùng và võng ở đó là gì? (CHỌN TẤT CẢ CÁC LOẠI MÀ HỌ CÓ)
Types of nets and hammocks (select all type they have)

- ☐ Mùng đơn
- ☐ Mùng đôi
- ☐ Mùng đơn tẩm thuốc MỀM
- ☐ Mùng đơn tẩm thuốc CỨNG
- ☐ Mùng đôi tẩm thuốc MỀM
- ☐ Mùng đôi tẩm thuốc CỨNG
- ☐ Mùng đơn tẩm thuốc tồn lưu MỀM
- ☐ Mùng đơn tẩm thuốc tồn lưu CỨNG
- ☐ Mùng đôi tẩm thuốc tồn lưu MỀM
- ☐ Mùng đôi tẩm thuốc tồn lưu CỨNG
- ☐ Võng không có mùng
- ☐ Võng có mùng không có khóa kéo
- ☐ Võng có mùng có khóa kéo
- ☐ Võng có mùng tẩm không có khóa kéo
- ☐ Võng có mùng tẩm có khóa kéo
- ☐ Võng có mùng tẩm tồn lưu không khóa kéo
- ☐ Võng có mùng tẩm tồn lưu có khóa kéo

- [ ] Võng không có mùng
- [ ] Võng có mùng không có khóa kéo
- [ ] Võng có mùng có khóa kéo
- [ ] Võng có mùng tẩm không có khóa kéo
- [ ] Võng có mùng tẩm có khóa kéo
- [ ] Võng có mùng tẩm tồn lưu không khóa kéo
- [ ] Võng có mùng tẩm tồn lưu có khóa kéo
- [ ] Khác

94. Nhóm của ông/bà có TỔNG SỐ bao nhiêu mùng ở nơi ngủ trong RẪY?
How many regular nets for sleepinggroup do you have?

____

95. Trong đó, Có bao nhiêu cái mùng được tẩm hóa chất?
How many regular nets treated for sleepinggroup?

____

96. Tổng cộng bao nhiêu cái võng tại nơi ngủ của nhóm ông/bà?
How many hammock for sleepinggroup do you have?

____

97. Trong đó, có bao nhiêu cái võng có mùng ở trong nhóm của ông/bà?
How many hammock with NET do sleepinggroup have?

____

98. Trong đó, có bao nhiêu cái võng mùng được tẩm hóa chất?
how many treated hammocks do you have in sleepinggroup?

____

99. Ở trong rẫy_Từ chòi/lều này tới rừng (bất kì) khoảng bao nhiêu Km? (Nếu trong rừng ghi số 0)
How far is your farm huts which is nearest to the forest from the farm?

____

100. Loại mùng nhóm ông/bà thích dùng NHẤT để ngủ CẢ ĐÊM trong RẪY là gì?
Type of net/hammock your workgroup like most to use

- ◯ Mùng đơn tẩm thuốc MỀM
- ◯ Mùng đơn tẩm thuốc CỨNG
- ◯ Mùng đôi tẩm thuốc MỀM
- ◯ Mùng đôi tẩm thuốc CỨNG
- ◯ Mùng đơn tẩm thuốc tồn lưu MỀM
- ◯ Mùng đơn tẩm thuốc tồn lưu CỨNG
- ◯ Mùng đôi tẩm thuốc tồn lưu MỀM
- ◯ Mùng đôi tẩm thuốc tồn lưu CỨNG
- ◯ Võng có mùng tẩm không có khóa kéo
- ◯ Võng có mùng tẩm có khóa kéo
- ◯ Võng có mùng tẩm tồn lưu không khóa kéo
- ◯ Võng có mùng tẩm tồn lưu có khóa kéo
- ◯ Võng có mùng tẩm không có khóa kéo
- ◯ Võng có mùng tẩm có khóa kéo
- ◯ Võng có mùng tẩm tồn lưu không khóa kéo
- ◯ Võng có mùng tẩm tồn lưu có khóa kéo
- ◯ Khác

101. Mô tả mùng/võng đó để biết chính xác nó như thế nào?
Describe net/hammock your workgroup like most

---

102. Nếu không có thích nhất, thì nhóm ông/bà thích dùng loại nào khác?
If don't have which like the most, what types other can acceptable?

- ○ Mùng đơn tẩm thuốc MỀM
- ○ Mùng đơn tẩm thuốc CỨNG
- ○ Mùng đôi tẩm thuốc MỀM
- ○ Mùng đôi tẩm thuốc CỨNG
- ○ Mùng đơn tẩm thuốc tồn lưu MỀM
- ○ Mùng đơn tẩm thuốc tồn lưu CỨNG
- ○ Mùng đôi tẩm thuốc tồn lưu MỀM
- ○ Mùng đôi tẩm thuốc tồn lưu CỨNG
- ○ Võng có mùng tẩm không có khóa kéo
- ○ Võng có mùng tẩm có khóa kéo
- ○ Võng có mùng tẩm tồn lưu không khóa kéo
- ○ Võng có mùng tẩm tồn lưu có khóa kéo
- ○ Võng có mùng tẩm không có khóa kéo
- ○ Võng có mùng tẩm có khóa kéo
- ○ Võng có mùng tẩm tồn lưu không khóa kéo
- ○ Võng có mùng tẩm tồn lưu có khóa kéo
- ○ Khác

103. Mô tả mùng/võng đó để biết chính xác nó như thế nào?
Describle net/hammock your workgroup like most

___

104. Ông bà có thích loại mùng tồn lưu cứng của Chương trình Quốc gia cấp không?
Would you like the polyethylen nets provided by National Program?

- ○ Có
- ○ Không

105. Nếu như không thích, ông bà cho biết lý do tại sao?
If no, Why you don't like?

___

106. Ông/bà có thích loại võng có mùng mà chương trình quốc gia cấp?
Would you like the hammock nets provided by National Program?

- ○ Có
- ○ Không

107. Nếu như không thích, ông bà cho biết lý do tại sao?
If no, Why you don't like?

___

108. Ông/bà có loại mùng/võng nào dưới đây Ở NHÀ? Đọc đáp án
Do you have net and/or hammock at home? Reading choices

- ☐ Mùng
- ☐ Võng có mùng
- ☐ Võng không có mùng nhưng có mùng kèm theo hoặc bọc võng
- ☐ Không có cả ba

109. Ông/bà có thể mang những mùng/võng có mùng này vào RẪY không?
Can you bring nets/hamock you have from home?

- ○ Có
- ○ Không

110. Nếu không mang Tại sao không?
Why not - consider list

- [ ] Không có do không tiền mua
- [ ] Không có do không biết chỗ mua
- [ ] Không có do xa quá không đi được để mua
- [ ] Nhà đang dùng không thể lấy đi
- [ ] Quá nặng để mang đi
- [ ] Khác

111. NHÓM NGỦ CHUNG với ông/bà có thể mang mùng đơn và/hoặc võng có màng CÓ Ở NHÀ KHI ĐI RẪY KHÔNG? (Dự đoán)
Sleepinggroup can bring single bednet or/and hammock with net WHICH SLEEPINGGROUP HAVE AT HOME go to FARM. (approximately)

- ○ Có
- ○ Không

112. Ở trong rẫy, Ông/bà có đi lấy nước sinh hoạt/ra ngoài sau khi trời tối hoặc trước khi trời sáng không?
Is patient going to get drinking water outside location after dark or before dawn?

- ○ Có
- ○ Không

113. Ở trong rẫy, Ông/bà có đi tắm ở suối/khe nước sau khi trời tối không?
Bathing outside house after dark

- ○ Có
- ○ Không

114. Ở trong rẫy, Ông/bà thường bị muỗi đốt khi làm gì - kể tất cả?
*What do you do when mosquitoes bother you - all_*

- [ ] Đi tắm
- [ ] Ăn cơm
- [ ] Nấu cơm
- [ ] Đang làm việc
- [ ] Ngồi nghỉ
- [ ] Ngủ

115. Ở trong rẫy, Làm gì thì bị muỗi cắn nhiều nhất
What do you do when mosquitoes bother you most - Main

- ○ Đi tắm
- ○ Ăn cơm
- ○ Nấu cơm
- ○ Đang làm việc
- ○ Ngồi nghỉ
- ○ Ngủ

116. Theo đánh giá của cán bộ điều tra, có bao nhiêu màn cần được tẩm cho nhóm ngủ trong RẪY này ?
In the interviewer's judgement, how many nets need to be treated?

____

117. Theo đánh giá của cán bộ điều tra, có cần mua mùng mới không cho nhóm ngủ trong RẪY này?
In the interviewer's judgement, do new nets need to be purchased?

- ○ Có

Không

118. Theo đánh giá của cán bộ điều tra, loại mùng nào có giá cả hợp lý nhất và được chấp nhận nhiều nhất (số lượng và loại) cho nhóm ngủ trong RẪY này? (Bao nhiêu cái và loại nào? Thêm giá vào mỗi cái mùng)

In the interviewer's judgement, what is the most affordable, acceptable solution (Number of nets and which type)?(Add cost of each net to the list)

____

119. Theo đánh giá của cán bộ điều tra, giải pháp nào được cho là hợp lý nhất để giảm trừ lây truyền sốt rét cho nhóm ngủ trong RẪY này? (số mùng và loại mùng mà nhóm này thực sự sẽ dùng)

In the interviewer's judgement, what is the best solution to reduce malaria transmission (Number of nets and which type that people will really use)?

____

120. Theo đánh giá của điều tra viên, Nếu loại màn cần mua có giá cao, nhóm ngủ trong RẪY này có sẵn sàng chi trả 1/2 giá tiền với dự án không? (có thể hỏi người dân để trả lời câu hỏi này)

In the interviewer's judgement, If the chosen type of net has high price, can people share half cost with Project?

Có

Không

NHÓM CHỈ ĐI RẪY

121. Ông/bà có muốn tẩm hóa chất cho mùng của ông/bà (chưa được tẩm) không?

Có

Không

122. Ông/bà có muốn tẩm hóa chất cho cả võng và mùng của võng ông/bà (chưa được tẩm) không?

Would you be willing to treat yourentirehammockandnet

Có

Không

123. Nếu không tẩm cả võng và mùng của võng, thì chỉ tẩm mùng của võng được không?

Would you be willing to treat just only the net of hammock?

Có

Không

124. Ông/bà có muốn dùng kem bôi chống muỗi không?
Would you be willing to use mosquito repellant ?

- Có
- Không

125. Ông/bà có muốn uống thuốc phòng sốt rét trước khi đi vào RẪY không? (UỐNG MỖI VIÊN MỘT NGÀY TRONG SUỐT THỜI GIAN Ở RỪNG)
Would you be willing to take medicine before you go to the FF in the future to prevent malaria? (one pill per day with meals while in forest)

- Có
- Không

126. Cách nào/phương tiện nào tốt nhất để tuyên truyền/giáo dục Ông/bà về sốt rét?
Which is thebest way toeducate people about malaria

____

127. Kiểu nhà của ông/bà là gì? (Có thể quan sát và đánh vào, không cần hỏi)
Type of housing structure (observation for weath)

- Nhà sàn
- Nhà trệt
- Khác

128. Mái nhà của ông/bà được làm bằng gì? (Có thể quan sát và đánh vào, không cần hỏi)
Roof type(observation for weath)

- Tre
- Gỗ
- Ngói
- Xi-măng
- Tôn

- ◯ Lá

129. Tường nhà của ông/bà làm bằng gì? (Có thể quan sát và đánh vào, không cần hỏi)

Wall type (observation for weath)

- ◯ Gạch
- ◯ Đất
- ◯ Tre
- ◯ Lá
- ◯ Sắt
- ◯ Khác
- ◯ Không có

130. Nhà vệ sinh của gia đình ông/bà là loại gì?
Toilet type (observation for weath)

- ◯ Nhà vệ sinh tự hoại
- ◯ Nhà vệ sinh khép kín
- ◯ Nhà vệ sinh không khép kín
- ◯ Không có nhà vệ sinh
- ◯ Khác

131. Nguồn nước chính gia đình ông/bà sử dụng là gì?
main water source

- Sông
- Suối
- Giếng đào
- Nước mưa
- Hồ/Ao
- Nước giếng khoan công cộng
- Nước máy
- Khác

132. Nguồn nước cách rừng bao xa? (Km)
Estimate KM

____

133. Nguồn nước chính gia đình ông/bà sử dụng trong MÙA KHÔ là gì?
main water source dry season

- Sông
- Suối
- Giếng/Giếng khoan
- Nước mưa
- Hồ/Ao
- Nước giếng khoan

- ◯ Khác

134. Nguồn nước cách rừng bao xa? (KM)
Estimate KM

____

135. Bạn có quan tâm đến việc hướng dẫn người khác biết đến phòng và điều trị sốt rét
Are you interested in teaching others about malaria prevention and elimination?

- ◯ Có
- ◯ Không
- ◯ Có thể

136. Người điều tra tự đánh giá: Người này có khả năng trở thành cộng tác viên được không?
Should we consider recruiting this person as a peer educator?

- ◯ Có
- ◯ Không
- ◯ Có thể

137. Tổng thu nhập mỗi năm của gia đình ông/bà là bao nhiêu? (Liệt kê tổng thu nhật từ rừng, chăn nuôi, thu nhập khác... Đơn vị tính triệu đồng)
Avg income year of family

____

138. Thu nhập hằng năm từ SẢN PHẨM TỪ rừng của gia đình là bao nhiêu? (Triệu đồng)
Income forest products

____

139. Thu nhập hằng năm từ làm việc tại rừng của gia đình là bao nhiêu? (Triệu đồng) (KỂ CẢ LÀM THUÊ Ở RỪNG)
Income forest labor

____

140. Gia đình ông/bà có bao nhiêu chiếc xe máy?
How many motocycle do you have?

141. Gia đình ông/bà có bao nhiêu chiếc TV?
How many television do you have?

142. Gia đình ông/bà có bao nhiêu chiếc đài radio?
How many radio do you have?

143. Số điện thoại ông/bà? (Lưu ý ghi số điện thoại của hộ gia đình)
Phone1

144. Nếu không có điện thoại, xin số điện thoại người thân hoặc ở gần nhà?
Phone2

145. Thôn/Bản
Village village

146. Xã
Commune

- ◯ Xuân Quang 1
- ◯ Xuân Lãnh
- ◯ Phú Mỡ
- ◯ Thanh
- ◯ Xy
- ◯ Ba Tầng

147. Tên phỏng vấn viên
Interviewer name

Interviewer name

148. Tên giám sát viên

Supervisior/Checked person name
